# Connection between Abundance of Food and Mortality

**Published:** 1842-03

**Authors:** 


*Connection between abundance of food and mortality.* By M. Melier.—In this memoir, which was read at the Academy of Medicine of Paris on the 7th of September, the author established, by numerous documents drawn from the histories of various countries, that the number of deaths always correspond to the price of food. "Wherever there's a loaf added there's a man born," said an economist; and nothing is more true than this metaphorical expression. If we represent the variations of the general mortality and those of the price of bread at different times, by two curved lines which rise and fall with all the fluctuations of these particulars, we shall find all their curvatures exactly, and with the most perfect regularity, corresponding. The constant increase of the population of France for a certain number of years, is easily explained by the progress of agriculture, the modifications which the laws relating to corn have undergone, and especially by the introduction of potatoes. The influence of the dearness of food, however, is observed more distinctly in the year next following than in any in which it has occurred.—*London Med. Gaz.*, from *Gaz. Med.*, September 10, 1841.

